# Incidence and risk factors associated with human albumin administration following total joint arthroplasty: a multicenter retrospective study

**DOI:** 10.1186/s13018-021-02642-9

**Published:** 2021-10-30

**Authors:** Shaoyun Zhang, Haibo Si, Jinwei Xie, Yuangang Wu, Qinsheng Hu, Yi Zeng, Fuxing Pei, Bin Shen

**Affiliations:** 1 Department of Orthopedics, Orthopedic Research Institute, West China Hospital, Sichuan University, No. 37 Guoxue Road, Chengdu, 610041 Sichuan Province People’s Republic of China; 2 Department of Orthopedics, The Third Hospital of Mianyang, Sichuan Mental Health Center, Mianyang, Sichuan Province People’s Republic of China

**Keywords:** Risk factor, Human albumin, Hypoalbuminemia, Total hip arthroplasty, Total knee arthroplasty

## Abstract

**Background:**

Enhanced recovery after surgery (ERAS) program advocates implementation of perioperative goal-directed fluid therapy and reduced application of colloidal fluids. It should be used reasonably selectively in high-risk patients despite the clear efficacy of human albumin (HA). Therefore, it is vital to identify the risk factors for the use of HA. This study aims to determine the incidence and risk factors of HA administration in patients undergoing total hip or knee arthroplasty (THA, TKA).

**Methods:**

We identified patients undergoing THA or TKA in multiple institutions from 2014 to 2016 and collected patient demographics and perioperative variables. The criterion of HA administration was defined as a postoperative albumin level < 32 g/L or 32 to 35 g/L for at-risk patients. We compared 14 variables between patients who received HA administration and those who did not after stratification by the preoperative albumin (pre-ALB) level. Multivariable regressions identified the independent risk factors associated with HA administration.

**Results:**

In total, 958 (20.3%) of 4713 patients undergoing THA and 410 (9.7%) of 4248 patients undergoing TKA received HA administration. In addition to pre-ALB < 35 g/L, preoperative anemia (odds ratio [OR] 2.12, *P* = 0.001; OR 1.39, *P* < 0.001) and drain use (OR 3.33, *P* = 0.001; OR 4.25, *P* < 0.001) were also independent risk factors for HA administration after THA regardless pre-ALB < 35 g/L or not, and patients undergoing TKA diagnosed of rheumatoid arthritis or ankylosing spondylitis tended to receive HA administration regardless pre-ALB < 35 g/L or not (OR 3.67, *P* = 0.002; OR 2.06, *P* < 0.001).

**Conclusions:**

The incidence of HA administration was high in patients undergoing THA or TKA, and several variables were risk factors for HA administration. This finding may aid surgeons in preoperatively identifying patients requiring HA administration and optimizing perioperative managements.

**Supplementary Information:**

The online version contains supplementary material available at 10.1186/s13018-021-02642-9.

## Introduction

Total joint arthroplasty (TJA) is a successful orthopedic procedure and the full numbers increase year by year [1]. Along with changes of the payment models, it is more and more necessary to reduce postoperative complications and shorten the length of stay [2]. In the last few years, the implementation of enhanced recovery after surgery (ERAS) program in the perioperative management of total hip arthroplasty (THA) or total knee arthroplasty (TKA) has been shown to be safe and feasible, with similar or better outcomes for the patients [3, 4].

The ERAS program is a series of perioperative multimodal strategies to enhance recovery and reduce morbidity, which advocates the application of perioperative goal-directed fluid therapy and reduced usage of colloidal fluids [5]. Human albumin (HA) is a colloid that can be used to treat hypoalbuminemia (< 35 g/L) and has been shown to increase postoperative albumin levels and reduce postoperative complications [6, 7]. However, it should be used reasonably selectively in those high-risk patients rather than unrestrained, because of its high cost and possible risk of anaphylaxis, renal insufficiency, and cardiac complications, especially for elderly patients undergoing TJA [8–10]. Therefore, it is the requirement of the modern ERAS program to identify high-risk patients and manage the application of HA efficiently.

A previous study found that female patients, with long operation times, and with low preoperative albumin (pre-ALB) levels were associated with a higher likelihood of HA administration, but the total sample size was too small and only 16 patients received HA administration [11]. Thus, this large multicenter study was performed to determine the real incidence of HA administration in patients undergoing THA or TKA. Furthermore, 14 independent variables were collected and identified to find the risk factors for HA administration in our study. It is hypothesized that patients with low pre-ALB levels undergoing THA or TKA were more likely to receive HA administration.

## Methods

### Ethics approval

This study was approved by the hospital’s institutional review board (2012-268), and the requirement for written informed consent was waived since the data are anonymized.

### Study design and participants

**Fig. 1:**
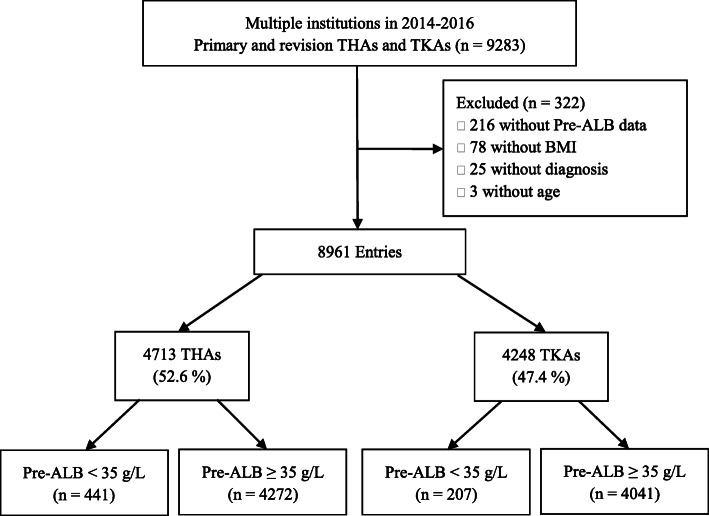
A flowchart shows the patient selection process. BMI, body mass index; Pre-ALB, preoperative albumin; THA, total hip arthroplasty; TKA, total knee arthroplasty

A retrospective analysis of data was collected on patients undergoing primary or revision THA or TKA in multiple institutions from 2014 to 2016 through the National Health Database [12]. Patients were excluded if they had missing data on preoperative serum albumin level or important demographic characteristics (Fig. 1).

The criterion of HA administration was determined by dietitians and surgeons, which was set as an albumin level on postoperative day 1 or 3 of < 32 g/L or 32 ~ 35 g/L but with lack of appetite or incision exudation [6]. According to postoperative albumin level, a single bolus of 10 g or 20 g of HA was administrated intravenously, and further infusion was decided after reexamination. In addition to HA administration, multimodal nutritional managements were also provided including oral carbohydrate-containing drink, oral protein powder, intravenous essential amino acid (EAA) supplementation, and oral gastric motility drugs or digestants [6, 13]. For patients with a postoperative albumin level of 35 ~ 40 g/L, they were encouraged to add high-protein diets such as eggs whenever possible.

After stratification by the surgical site (THA or TKA), patients were grouped into those with pre-ALB < 35 g/L and those with pre-ALB ≥ 35 g/L. The potential variables associated with HA administration including patient demographic characteristics such as age, sex, and body mass index (BMI), and perioperative factors were all collected and analyzed. Perioperative factors included diagnosis, American Society of Anesthesiologists (ASA) class, preoperative anemia, surgical type, anesthesia, tourniquet use (for TKA), anticoagulant use, tranexamic acid use, colloid solution use, drain use, and transfusion use.

### Statistical analysis

Pearson chi-square test or Fisher’s exact test was used for categorical variables to assess the differences of proportions between the HA and non-HA groups. A forward LR model for multivariable logistic regression analysis was used to examine independent risk factors for HA administration. ORs, corresponding 95% CIs, and *p* values were computed. A *p* value of < 0.05 was considered statistically significant. Due to 14 clinical variables were evaluated, a conservative Bonferroni *p* value was computed as 0.05/14 for the significance of multivariable regression [14]. All analyses were performed using IBM SPSS version 24.0.

## Results

Of 9283 initial patients, 8961 (96.5%) met inclusion criteria (Fig. 1). Among them, 4713 (52.6%) patients undergoing THA, whereas 4248 (47.4%) undergoing TKA were included. There were 958 (20.3%) patients received HA administration and 441 (9.4%) patients with pre-ALB < 35 g/L in THA group, while 410 (9.7%) patients received HA administration and 207 (4.9%) patients with pre-ALB < 35 g/L in TKA group.

The results of the comparison of demographic characteristics and perioperative factors between HA and non-HA groups in patients undergoing THA or TKA are shown in Supplemental Tables 1 and 2, which were greatly influenced by differences in pre-ALB levels. Thus, after stratified by pre-ALB levels, we compared those again in these patients. For patients undergoing THA, HA administration was associated with diagnosis, preoperative anemia, anesthesia, anticoagulant use, and drain use in those with pre-ALB < 35 g/L (*P* < 0.05), which was associated with age, sex, diagnosis, ASA class, preoperative anemia, surgical type, anesthesia, anticoagulant use, colloid solution use, and drain use in those with pre-ALB ≥ 35 g/L (*P* < 0.05) (Table 1). For patients undergoing TKA, HA administration was associated with BMI and diagnosis in those with pre-ALB < 35 g/L (*P* < 0.05), which was associated with diagnosis, preoperative anemia, surgical type, anesthesia, colloid solution use, and drain use in those with pre-ALB ≥ 35 g/L (*P* < 0.05) (Table 2).

**Table 1:** Comparison of demographic characteristics and perioperative factors between HA and non-HA groups in patients undergoing THA after stratified by preoperative serum albumin levels*

Variable	Pre-ALB < 35 g/L				Pre-ALB ≥ 35 g/L			
	Overall (*n* = 441)	HA (*n* = 297)	No HA (*n* = 144)	*P* value†	Overall (*n* = 4272)	HA (*n* = 661)	No HA (*n* = 3611)	*P* value†
Age (years)				0.593				0.001
≤ 64	253 (57.4)	175 (69.2)	78 (30.8)		2932 (68.6)	414 (14.1)	2518 (85.9)	
65–79	137 (31.1)	90 (65.7)	47 (34.3)		1194 (27.9)	219 (18.3)	975 (81.7)	
≥ 80	51 (11.6)	32 (62.7)	19 (37.3)		146 (3.4)	28 (19.2)	118 (80.8)	
Sex				0.069				0.019
Male	205 (46.5)	147 (71.7)	58 (28.3)		2015 (47.2)	284 (14.1)	1731 (85.9)	
Female	236 (53.5)	150 (63.6)	86 (36.4)		2257 (52.8)	377 (16.7)	1880 (83.3)	
BMI (kg/m^2^)				0.084				0.120
< 18.5	45 (10.2)	31 (68.9)	14 (31.1)		229 (5.4)	46 (20.1)	183 (79.9)	
18.5–24.9	281 (63.7)	178 (63.3)	103 (36.7)		2639 (61.8)	412 (15.6)	2227 (84.4)	
25.0–29.9	103 (23.4)	78 (75.7)	25 (24.3)		1184 (27.7)	176 (14.9)	1008 (85.1)	
≥ 30	12 (2.7)	10 (83.3)	2 (16.7)		220 (5.1)	27 (12.3)	193 (87.7)	
Diagnosis				0.008				0.001
ONFH	157 (35.6)	108 (68.8)	49 (31.2)		1420 (33.2)	179 (12.6)	1241 (87.4)	
DDH	25 (5.7)	16 (64.0)	9 (36.0)		842 (19.7)	125 (14.8)	717 (85.2)	
OA	55 (12.5)	48 (87.3)	7 (12.7)		872 (20.4)	161 (18.5)	711 (81.5)	
HF	110 (24.9)	66 (60.0)	44 (40.0)		394 (9.2)	69 (17.5)	325 (82.5)	
Others	94 (21.3)	59 (62.8)	35 (37.2)		744 (17.4)	127 (17.1)	617 (82.9)	
ASA class				0.075				< 0.001
1	169 (38.3)	103 (60.9)	66 (39.1)		1471 (34.4)	168 (11.4)	1303 (88.6)	
2	226 (51.2)	162 (71.7)	64 (28.3)		2461 (57.6)	430 (17.5)	2031 (82.5)	
≥ 3	46 (10.4)	32 (69.6)	14 (30.4)		340 (8.0)	63 (18.5)	277 (81.5)	
Anemia				0.002				< 0.001
Yes	212 (48.1)	158 (74.5)	54 (25.5)		1725 (40.4)	318 (18.4)	1407 (81.6)	
No	229 (51.9)	139 (60.7)	90 (39.3)		2547 (59.6)	343 (13.5)	2204 (86.5)	
Surgical type				0.835				0.024
Primary unilateral	388 (88.0)	263 (67.8)	125 (32.2)		3873 (90.7)	602 (15.5)	3271 (84.5)	
Primary bilateral	32 (7.3)	21 (65.6)	11 (34.4)		221 (5.2)	23 (10.4)	198 (89.6)	
Revision unilateral	21 (4.8)	13 (61.9)	8 (38.1)		178 (4.2)	36 (20.2)	142 (79.8)	
Anesthesia				0.028				0.001
General	373 (84.6)	259 (69.4)	114 (30.6)		3635 (85.1)	591 (16.3)	3044 (83.7)	
Spinal + epidural + CSE	68 (15.4)	38 (55.9)	30 (44.1)		637 (14.9)	70 (11.0)	567 (89.0)	
Anticoagulant use				0.040				0.043
Yes	418 (94.8)	286 (68.4)	132 (31.6)		4094 (95.8)	643 (15.7)	3451 (84.3)	
No	23 (5.2)	11 (47.8)	12 (52.2)		178 (4.2)	18 (10.1)	160 (89.9)	
TXA use				0.758				0.492
Yes	167 (37.9)	111 (66.5)	56 (33.5)		2771 (64.9)	421 (15.2)	2350 (84.8)	
No	274 (62.1)	186 (67.9)	88 (32.1)		1501 (35.1)	240 (16.0)	1261 (84.0)	
Colloid solution use				0.173				< 0.001
Yes	292 (66.2)	203 (69.5)	89 (30.5)		2349 (55.0)	418 (17.8)	1931 (82.2)	
No	149 (33.8)	94 (63.1)	55 (36.9)		1923 (45.0)	243 (12.6)	1680 (87.4)	
Drain use				0.002				< 0.001
Yes	405 (91.8)	281 (69.4)	124 (30.6)		3516 (82.3)	624 (17.7)	2892 (82.3)	
No	36 (8.2)	16 (44.4)	20 (55.6)		756 (17.7)	37 (4.9)	719 (95.1)	
Transfusion use				0.970				0.251
Yes	77 (17.5)	52 (67.5)	25 (32.5)		792 (18.5)	112 (14.1)	680 (85.9)	
No	364 (82.5)	245 (67.3)	119 (32.7)		3480 (81.5)	549 (15.8)	2931 (84.2)	

*Data are reported as number (%); †p-value calculated using Pearson chi-square test or Fisher exact test

*ASA* American Society of Anesthesiologists, *BMI* body mass index, *CSE* combined spinal-epidural, *DDH* development dysplasia of hip, *HA* human albumin, *HF* hip fracture, *OA* osteoarthritis, *ONFH* osteonecrosis of femoral head, *Pre-ALB* preoperative albumin, *THA* total hip arthroplasty, *TXA* tranexamic acid

**Table 2:** Comparison of demographic characteristics and perioperative factors between HA and non-HA groups in patients undergoing TKA after stratified by preoperative serum albumin levels*

Variable	Pre-ALB < 35 g/L				Pre-ALB ≥ 35 g/L			
	Overall (*n* = 207)	HA (*n* = 127)	No HA (*n* = 80)	*P* value†	Overall (*n* = 4041)	HA (*n* = 283)	No HA (*n* = 3758)	*P* value†
Age (years)				0.471				0.205
≤ 64	79 (38.2)	50 (63.3)	29 (36.7)		1708 (42.3)	108 (6.3)	1600 (93.7)	
65–79	113 (54.6)	70 (61.9)	43 (38.1)		2185 (54.1)	167 (7.6)	2018 (92.4)	
≥ 80	15 (7.2)	7 (46.7)	8 (53.3)		148 (3.7)	8 (5.4)	140 (94.6)	
Sex				0.376				0.139
Male	59 (28.5)	39 (66.1)	20 (33.9)		792 (19.6)	65 (8.2)	727 (91.8)	
Female	148 (71.5)	88 (59.5)	60 (40.5)		3249 (80.4)	218 (6.7)	3031 (93.3)	
BMI (kg/m^2^)				0.002				0.115
< 18.5	13 (6.3)	9 (69.2)	4 (30.8)		74 (1.8)	7 (9.5)	67 (90.5)	
18.5–24.9	103 (49.8)	59 (57.3)	44 (42.7)		1637 (40.5)	125 (7.6)	1512 (92.4)	
25.0–29.9	67 (32.4)	36 (53.7)	31 (38.8)		1753 (43.4)	104 (5.9)	1649 (94.1)	
≥ 30	24 (11.6)	23 (95.8)	1 (4.2)		577 (14.3)	47 (8.1)	530 (91.9)	
Diagnosis				0.008				< 0.001
OA	158 (76.3)	88 (55.7)	70 (44.3)		3678 (91.0)	229 (6.2)	3449 (93.8)	
RA + AS	41 (19.8)	32 (78.0)	9 (22.0)		252 (6.2)	32 (12.7)	220 (87.3)	
Others	8 (3.9)	7 (87.5)	1 (12.5)		111 (2.7)	22 (19.8)	89 (80.2)	
ASA class				0.092				0.382
1	74 (35.7)	52 (70.3)	22 (29.7)		1660 (41.1)	106 (6.4)	1554 (93.6)	
2	110 (53.1)	60 (54.5)	50 (45.5)		2087 (51.6)	153 (7.3)	1934 (92.7)	
≥ 3	23 (11.1)	15 (65.2)	8 (34.8)		294 (7.3)	24 (8.2)	270 (91.8)	
Anemia				0.338				0.012
Yes	77 (37.2)	44 (57.1)	33 (42.9)		1111 (27.5)	96 (8.6)	1015 (91.4)	
No	130 (62.8)	83 (63.8)	47 (36.2)		2930 (72.5)	187 (6.4)	2743 (93.6)	
Surgical type				0.060				0.035
Primary unilateral	159 (76.8)	92 (57.9)	67 (42.1)		3503 (86.7)	257 (7.3)	3246 (92.7)	
Primary bilateral	42 (20.3)	32 (76.2)	10 (23.8)		494 (12.2)	22 (4.5)	472 (95.5)	
Revision unilateral	6 (2.9)	3 (50.0)	3 (50.0)		44 (1.1)	4 (9.1)	40 (90.9)	
Anesthesia				0.281				0.040
General	151 (72.9)	96 (63.6)	55 (36.4)		2883 (71.3)	217 (7.5)	2666 (92.5)	
Spinal + epidural + CSE	56 (27.1)	31 (55.4)	25 (44.6)		1158 (28.7)	66 (5.7)	1092 (94.3)	
Tourniquet use				0.401				0.544
Yes	181 (87.4)	113 (62.4)	68 (37.6)		3316 (82.1)	236 (7.1)	3080 (92.9)	
No	26 (12.6)	14 (53.8)	12 (46.2)		725 (17.9)	47 (6.5)	678 (93.5)	
Anticoagulant use				0.566				0.703
Yes	194 (93.7)	120 (61.9)	74 (38.1)		3881 (96.0)	273 (7.0)	3608 (93.0)	
No	13 (6.3)	7 (53.8)	6 (46.2)		160 (4.0)	10 (6.3)	150 (93.7)	
TXA use				0.652				0.369
Yes	102 (49.3)	61 (59.8)	41 (40.2)		2832 (70.1)	205 (7.2)	2627 (92.8)	
No	105 (50.7)	66 (62.9)	39 (37.1)		1209 (29.9)	78 (6.5)	1131 (93.5)	
Colloid solution use				0.822				0.005
Yes	157 (75.8)	97 (61.8)	60 (38.2)		2198 (54.4)	131 (6.0)	2067 (94.0)	
No	50 (24.2)	30 (60.0)	20 (40.0)		1843 (45.6)	152 (8.2)	1691 (91.8)	
Drain use				0.381				< 0.001
Yes	174 (84.1)	109 (62.6)	65 (37.4)		3455 (85.5)	265 (7.7)	3190 (92.3)	
No	33 (15.9)	18 (54.5)	15 (45.5)		586 (14.5)	18 (3.1)	568 (96.9)	
Transfusion use				0.124				0.199
Yes	36 (17.4)	18 (50.0)	18 (50.0)		789 (19.5)	47 (6.0)	742 (94.0)	
No	171 (82.6)	109 (63.7)	62 (36.3)		3252 (80.5)	236 (7.3)	3016 (92.7)	

*Data are reported as number (%); †p-value calculated using Pearson chi-square test or Fisher exact test

*AS* ankylosing spondylitis, *ASA* American Society of Anesthesiologists, *BMI* body mass index, *CSE* combined spinal-epidural, *HA* human albumin, *OA* osteoarthritis, *Pre-ALB* preoperative albumin, *RA* rheumatoid arthritis, *TKA* total knee arthroplasty, *TXA* tranexamic acid

**Fig. 2:**
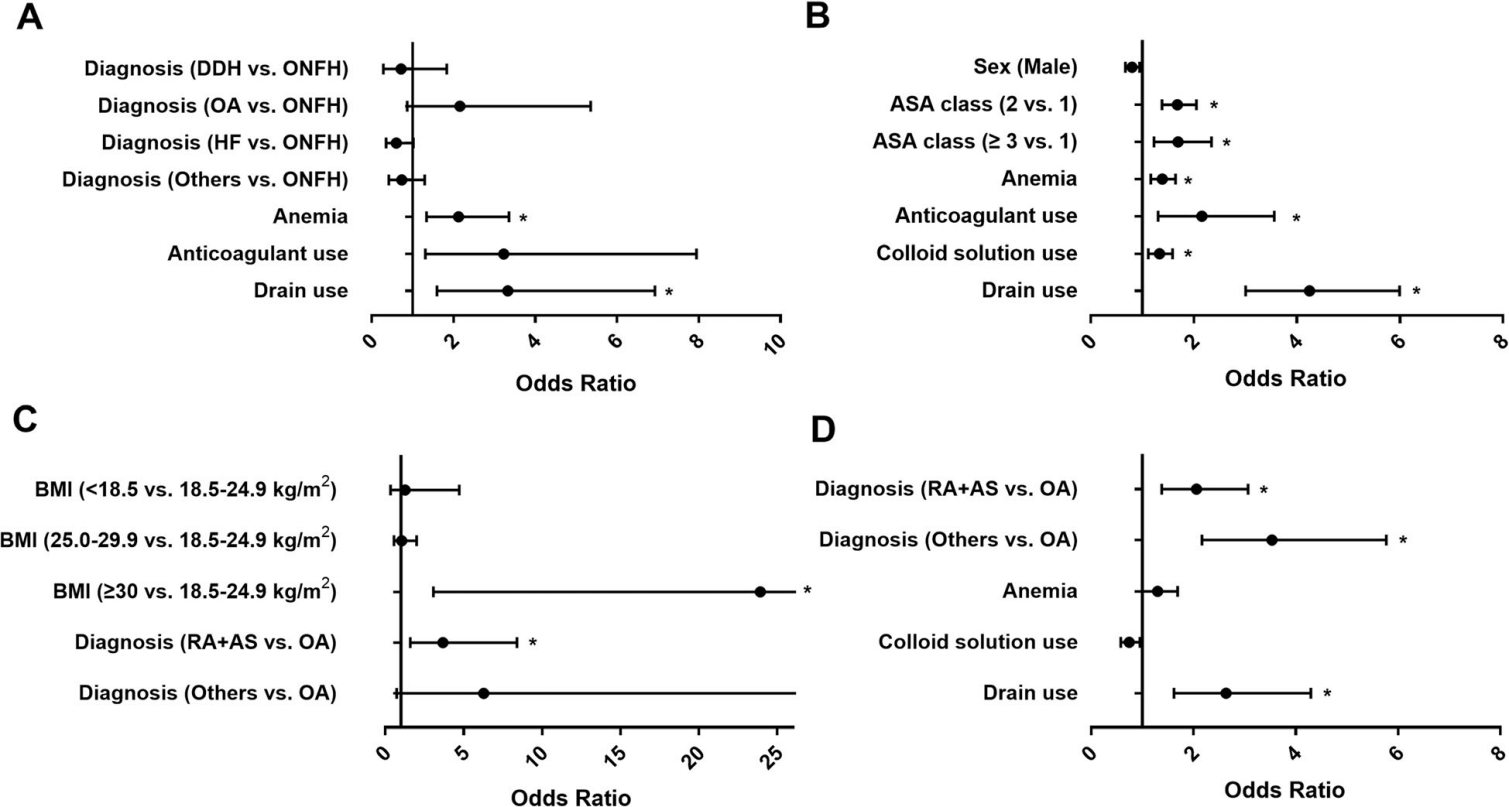
**A**–**D** Multivariable regression analysis of studied risk factors associated with human albumin administration in patients undergoing THA with Pre-ALB < 35 g/L (**A**), patients undergoing THA with Pre-ALB ≥ 35 g/L (**B**), patients undergoing TKA with Pre-ALB < 35 g/L (**C**), and patients undergoing TKA with Pre-ALB ≥ 35 g/L (**D**). The results are shown as odds ratios with 95% confidence intervals. * indicates a significant difference after Bonferroni correction. AS, ankylosing spondylitis; ASA**,** American Society of Anesthesiologists; BMI, body mass index; DDH, development dysplasia of hip; HF, hip fracture; OA, osteoarthritis; ONFH, osteonecrosis of femoral head; Pre-ALB, preoperative albumin; RA, rheumatoid arthritis; THA, total hip arthroplasty; TKA, total knee arthroplasty

After that, multivariable logistic regression analyses were conducted to identify independent risk factors for HA administration in patients undergoing THA with pre-ALB < 35 g/L (Fig. 2A), patients undergoing THA with pre-ALB ≥ 35 g/L (Fig. 2B), patients undergoing TKA with pre-ALB < 35 g/L (Fig. 2C), and patients undergoing TKA with pre-ALB ≥ 35 g/L (Fig. 2D). Preoperative anemia (OR 2.12, 95% CI 1.34–3.36, *P* = 0.001; OR 1.39, 95% CI 1.17–1.65, *P* < 0.001) and drain use (OR 3.33, 95% CI 1.60–6.93, *P* = 0.001; OR 4.25, 95% CI 3.01–5.99, *P* < 0.001) were significant risk factors for HA administration following THA regardless pre-ALB < 35 g/L or ≥ 35 g/L, while ASA class 2 (vs. 1, OR 1.68, 95% CI 1.38–2.05, *P* < 0.001) or 3 (vs. 1, OR 1.70, 95% CI 1.23–2.34, *P* = 0.001), anticoagulant use (OR 2.16, 95% CI 1.31–3.56, *P* = 0.003), and colloid solution use (OR 1.33, 95% CI 1.12–1.59, *P* = 0.001) were also significant risk factors for HA administration in patients undergoing THA with pre-ALB ≥ 35 g/L.

For patients undergoing TKA, diagnosis of rheumatoid arthritis (RA) or ankylosing spondylitis (AS) was independent risk factor for HA administration regardless pre-ALB < 35 g/L or ≥ 35 g/L when compared with those patients with osteoarthritis (OR 3.67, 95% CI 1.60–8.40, *P* = 0.002; OR 2.06, 95% CI 1.38–3.07, *P* < 0.001). Patients with BMI ≥ 30 kg/m^2^ (OR 23.91, 95% CI 3.07–186.02, *P* = 0.002) tended to receive HA administration than those with BMI between 18.5 and 24.9 kg/m^2^ in pre-ALB < 35 g/L group, whereas patients with drain use (OR 2.64, 95% CI 1.62–4.30, *P* < 0.001) tended to receive HA administration in pre-ALB ≥ 35 g/L group.

## Discussion

The most important finding of this large, multicenter, retrospective study was that the incidence of HA administration in patients undergoing THA or TKA was high, and several independent variables were risk factors for HA administration such as low pre-ALB levels, preoperative anemia, and drain use. To our knowledge, our study is the largest to date evaluating the independent risk factors associated with HA administration after THA or TKA. We focused specifically on variables of modifiable perioperative factors and demographic characteristics which help identify high-risk populations. In addition, we used stratification analysis to eliminate potential contributions of pre-ALB level on other variables in the multivariable regression models. Because of this analysis, we believe there is an association between these discovered risk factors and HA administration after THA or TKA.

Numerous studies have confirmed that hypoalbuminemia is an independent predictor of complications after THA and TKA [15–17], and HA administration is an effective way to improve ALB levels and correct hypoalbuminemia in a short term [7, 18]. Patients undergoing THA or TKA are often elderly, with or without malnutrition [15, 17], and they would be advised to receive HA administration when there is swelling or exudation of the wound due to hypoalbuminemia [6, 7]. However, this may not conform to the requirements of modern ERAS, which advocates the application of perioperative goal-directed fluid therapy and reduced usage of colloidal fluids [4, 5]. Moreover, patients are advocated to increase oral diet and restore gastrointestinal peristalsis and ambulation as soon as possible [3, 19]. Therefore, rational administration of HA is necessary. The purpose of this study is to clarify the current HA administration under the modern ERAS program and to reduce and standardize HA administration by identifying potentially high-risk patients and providing early intervention. In the current study, the incidence of HA administration in patients undergoing THA was 20.3%, while that was 9.7% in the TKA group. These were higher than that in Wu et al.’s study [11], which was only 4.1% in patients undergoing primary elective THA. This indicates that there is still a lot of work that needs to do to reduce the application of HA, including the formulation of strict administration guidelines [20], identification of high-risk patients and early intervention [11], shortening perioperative time of fasting and water-deprivation [13], and strengthening perioperative nutritional support [6].

In the current study, the pre-ALB level was still the most important risk factor for HA administration, which can be modified by a series of interventions. Cao et al. [6] conducted a randomized controlled trial in which 162 patients undergoing primary TKA were recruited to receive either a new multimodal nutritional management or a traditional protocol. The multimodal nutritional management included multiple nutrition and protein powder before and after surgery, and it resulted in a lower rate and amount of HA administration and shorter length of hospital stay. Moreover, in a similar prospective study that evaluated the effectiveness of perioperative essential amino acid supplementation, Ueyama et al. [21] demonstrated a significant increase in serum albumin with essential amino acid supplementation compared with placebo. A systematic review on the role of nutritional supplements in support of THA and TKA by Burgess et al. [22] concluded that optimizing nutritional status preoperatively may help manage the surgical stress response, with a particular benefit for undernourished, frail, or elderly individuals. Therefore, for patients with low pre-ALB levels, measures should be taken to optimize nutritional status, rather than just relying on HA administration.

The use of drain has been controversial in THA or TKA, with some advocated the use of drain because it reduced ecchymosis and avoided hematoma formation [23], while others had opposed the usage because it increased blood loss and prolonged postoperative length of stay [24]. In the current study, we found that the use of drain was an independent risk factor for HA administration after THA or TKA. This founding was failed in the group of patients undergoing TKA with pre-ALB < 35 g/L due to the small sample size. However, there is no doubt that the use of drain would lead to an increased risk of HA administration because theoretically, drain use leads to the loss of proteins and nutrients while extracting the hemorrhage, which is disadvantageous to wound recovery [25]. Therefore, the use of drain during THA or TKA should be avoided or removed earlier since prolonged use is not necessary [19].

Preoperative anemia has been found to be a risk factor for blood transfusion after THA or TKA [14] and increases the cost [26], but little has confirmed the association between anemia and HA administration. In the current study, we found that preoperative anemia was an independent modifiable risk factor for HA administration after THA, regardless of pre-ALB < 35 g/L or not, which has been confirmed before that anemia was associated with hypoalbuminemia [27]. In addition, we found that the incidence of anemia in patients undergoing THA was higher than those undergoing TKA, this may be related to that patients with hip fractures were included in the current study, because these patients were found to be with higher incidences of preoperative anemia, postoperative hypoalbuminemia, and HA administration [28]. Therefore, for patients undergoing THA with preoperative anemia, especially those with hip fractures, more attention should be paid to correct their anemia and nutritional status.

In the current study, we found that patients with inflammatory arthritis such as RA and AS tended to receive HA administration after TKA. Previous studies reported that active RA or AS patients were characterized by anemia and hypoalbuminemia [29, 30]. This may be associated with inflammation mediated by tumor necrosis factor-α and interleukin-6, which decreased the expression of the albumin gene at the transcriptional level in the liver [31]. Another explanation may be that the permeability of the blood-joint barrier for albumin in RA patients is markedly increased, resulting in high albumin uptake at sites of inflammation [32]. This gives us a reminder for clinical work, that is, patients with RA or AS should pay more attention to nutritional status and albumin level, once hypoalbuminemia occurs, do an early intervention.

For patients undergoing THA with pre-ALB ≥ 35 g/L, ASA class 2 or 3, anticoagulant use, and colloid solution use were also significant risk factors for HA administration after surgery. Patients with ASA classes 2 or 3 usually have more comorbidities, which have been shown to be strongly associated with low postoperative albumin levels [33]. The use of anticoagulants may cause more hidden blood loss [34], leading to lower postoperative albumin levels. In contrast, revision surgery was not a risk factor for HA administration for either THA or TKA. It may be related to the small sample size in the current study. Besides, for patients undergoing revision surgery, the requirements were more strictly for patient screening and improvement of comorbidities, which may be one reason for no increased risk of HA administration after surgery of these patients.

There are several limitations in the current study. First, due to the small sample size, we should be cautious when understanding the positive result of BMI ≥ 30 kg/m^2^ as a risk factor of HA administration in the group of patients undergoing TKA with pre-ALB < 35 g/L, although malnutrition increased when the rates of obesity increased [33]. Second, patients in the current study were grouped into those with pre-ALB < 35 g/L and those with pre-ALB ≥ 35 g/L, while patients were categorized into four groups: < 35 g/L, 35 to < 40 g/L, 40 to < 45 g/L, and ≥ 45 g/L by Rudasill et al. [8], and patients with hypoalbuminemia were categorized into quartiles: < 30 g/L, 30 to 31.9 g/L, 32 to 33.9 g/L, and 34 to 34.9 g/L by Kishawi et al. [33]. Therefore, we cannot find the association between the albumin gradient and HA administration like other studies. Third, our study was limited by the number of available variables in the national database. The postoperative albumin levels were not routinely recorded in the database, while the incidence of HA administration was carefully recorded and easily collected. However, the decision of HA administration was not objective enough and the criteria were not very strict, this may bias our results. Besides, the comparability of our results with other studies may be affected by the possible differences in HA administration among countries and regions. Finally, the study design was retrospective and observational, despite prospective data collection from the database. Prospective studies may be indicated to further confirm our findings.

## Conclusions

In conclusion, the incidence of HA administration in patients undergoing THA or TKA was high, and pre-ALB < 35 g/L and use of drain were modifiable risk factors for HA administration for patients undergoing either THA or TKA, while preoperative anemia, ASA class 2 or 3, anticoagulant use, and colloid solution use were independent modifiable risk factors for HA administration after THA, and patients with RA or AS were high-risk population for receiving HA administration after TKA. Our predictive model based on available patient demographic characteristics and modifiable perioperative factors may aid surgeons in preoperatively identifying patients requiring HA administration and optimizing perioperative managements.

## Supplementary Information

**Additional file 1.** Supplemental Table 1 Comparison of demographic characteristics and perioperative factors between HA and non-HA groups in patients undergoing THA*.

**Additional file 2.** Supplemental Table 2. Comparison of demographic characteristics and perioperative factors between HA and non-HA groups in patients undergoing TKA*.

## Data Availability

Please contact the author for data requests.

## Abbreviations

AS: Ankylosing spondylitis
ASA: American Society of Anesthesiologists
BMI: Body mass index
CI: Confidence interval
ERAS: Enhanced recovery after surgery
HA: Human albumin
OR: Odds ratio
Pre-ALB: Preoperative albumin
RA: Rheumatoid arthritis
THA: Total hip arthroplasty
TJA: Total joint arthroplasty
TKA: Total knee arthroplasty
