# Supplementary material for: Incidence and risk factors associated with human albumin administration following total joint arthroplasty: a multicenter retrospective study
Source: J Orthop Surg Res. 2021 Oct 30;16:643. doi: 10.1186/s13018-021-02642-9 (PMC8557000; doi:10.1186/s13018-021-02642-9)
Supplement: Supplementary file 1 — Additional file 1. Supplemental Table 1 Comparison of demographic characteristics and perioperative factors between HA and non-HA groups in patients undergoing THA*. [file 13018_2021_2642_MOESM1_ESM.doc]

Supplemental Table 1

Comparison of demographic characteristics and perioperative factors between HA and non-HA groups in patients undergoing THA*.

| Variable | Overall (n = 4713) | HA (n = 958) | No HA (n = 3755) | P-value† |
| --- | --- | --- | --- | --- |
| Age (yrs) |  |  |  | < 0.001 |
| ≤ 64 | 3185 (67.6) | 589 (18.5) | 2596 (81.5) |  |
| 65-79 | 1331 (28.2) | 309 (23.2) | 1022 (76.8) |  |
| ≥ 80 | 197 (4.2) | 60 (30.5) | 137 (69.5) |  |
| Sex |  |  |  | 0.142 |
| Male | 2220 (47.1) | 431 (19.4) | 1789 (80.6) |  |
| Female | 2493 (52.9) | 527 (21.1) | 1966 (78.9) |  |
| BMI (kg/m^2^) |  |  |  | 0.004 |
| < 18.5 | 274 (5.8) | 77 (28.1) | 197 (71.9) |  |
| 18.5-24.9 | 2920 (62.0) | 590 (20.2) | 2330 (79.8) |  |
| 25.0-29.9 | 1287 (27.3) | 254 (19.7) | 1033 (80.3) |  |
| ≥ 30 | 232 (4.9) | 37 (15.9) | 195 (84.1) |  |
| Diagnosis |  |  |  | < 0.001 |
| ONFH | 1577 (33.5) | 287 (18.2) | 1290 (81.8) |  |
| DDH | 867 (18.4) | 141 (16.3) | 726 (83.7) |  |
| OA | 927 (19.7) | 209 (22.5) | 718 (77.5) |  |
| HF | 504 (10.7) | 135 (26.8) | 369 (73.2) |  |
| Others | 838 (17.8) | 186 (22.2) | 652 (77.8) |  |
| ASA class |  |  |  | < 0.001 |
| 1 | 1640 (34.8) | 271 (16.5) | 1369 (83.5) |  |
| 2 | 2687 (57.0) | 592 (22.0) | 2095 (78.0) |  |
| ≥ 3 | 386 (8.2) | 95 (24.6) | 291 (75.4) |  |
| Anemia |  |  |  | < 0.001 |
| Yes | 1937 (41.1) | 476 (24.6) | 1461 (75.4) |  |
| No | 2776 (58.9) | 482 (17.4) | 2294 (82.6) |  |
| Pre-ALB (g/L) |  |  |  | < 0.001 |
| < 35 | 441 (9.4) | 297 (67.3) | 144 (32.7) |  |
| ≥ 35 | 4272 (90.6) | 661 (15.5) | 3611 (84.5) |  |
| Surgical type |  |  |  | 0.164 |
| Primary unilateral | 4261 (90.4) | 865 (20.3) | 3396 (79.7) |  |
| Primary bilateral | 253 (5.4) | 44 (17.4) | 209 (82.6) |  |
| Revision unilateral | 199 (4.2) | 49 (24.6) | 150 (75.4) |  |
| Anesthesia |  |  |  | < 0.001 |
| General | 4008 (85.0) | 850 (21.2) | 3158 (78.8) |  |
| Spinal + epidural + CSE | 705 (15.0) | 108 (15.3) | 597 (84.7) |  |
| Anticoagulant use |  |  |  | 0.034 |
| Yes | 4512 (95.7) | 929 (20.6) | 3583 (79.4) |  |
| No | 201 (4.3) | 29 (14.4) | 172 (85.6) |  |
| TXA use |  |  |  | < 0.001 |
| Yes | 2938 (62.3) | 532 (18.1) | 2406 (81.9) |  |
| No | 1775 (37.7) | 426 (24.0) | 1349 (76.0) |  |
| Colloid solution use |  |  |  | < 0.001 |
| Yes | 2641 (56.0) | 621 (23.5) | 2020 (76.5) |  |
| No | 2072 (44.0) | 337 (16.3) | 1735 (83.7) |  |
| Drain use |  |  |  | < 0.001 |
| Yes | 3921 (83.2) | 905 (23.1) | 3016 (76.9) |  |
| No | 792 (16.8) | 53 (6.7) | 739 (93.3) |  |
| Transfusion use |  |  |  | 0.238 |
| Yes | 869 (18.4) | 164 (18.9) | 705 (81.1) |  |
| No | 3844 (81.6) | 794 (20.7) | 3050 (79.3) |  |

*Data are reported as number (%); †p-value calculated using Pearson chi-square test.

ASA**,** American Society of Anesthesiologists; BMI, body mass index; CSE, combined spinal-epidural; DDH, development dysplasia of hip; HA, human albumin; HF, hip fracture; OA, osteoarthritis; ONFH, osteonecrosis of femoral head; Pre-ALB, preoperative albumin; THA, total hip arthroplasty; TXA, tranexamic acid.
