# Supplementary material for: Incidence and risk factors associated with human albumin administration following total joint arthroplasty: a multicenter retrospective study
Source: J Orthop Surg Res. 2021 Oct 30;16:643. doi: 10.1186/s13018-021-02642-9 (PMC8557000; doi:10.1186/s13018-021-02642-9)
Supplement: Supplementary file 2 — Additional file 2. Supplemental Table 2. Comparison of demographic characteristics and perioperative factors between HA and non-HA groups in patients undergoing TKA*. [file 13018_2021_2642_MOESM2_ESM.doc]

Supplemental Table 2

Comparison of demographic characteristics and perioperative factors between HA and non-HA groups in patients undergoing TKA*.

| Variable | Overall (n = 4248) | HA (n = 410) | No HA (n = 3838) | P-value† |
| --- | --- | --- | --- | --- |
| Age (yrs) |  |  |  | 0.281 |
| ≤ 64 | 1787 (42.1) | 158 (8.8) | 1629 (91.2) |  |
| 65-79 | 2298 (54.1) | 237 (10.3) | 2061 (89.7) |  |
| ≥ 80 | 163 (3.8) | 15 (9.2) | 148 (90.8) |  |
| Sex |  |  |  | 0.005 |
| Male | 851 (20.0) | 104 (12.2) | 747 (87.8) |  |
| Female | 3397 (80.0) | 306 (9.0) | 3091 (91.0) |  |
| BMI (kg/m^2^) |  |  |  | < 0.001 |
| < 18.5 | 87 (2.0) | 16 (18.4) | 71 (81.6) |  |
| 18.5-24.9 | 1740 (41.0) | 184 (10.6) | 1556 (89.4) |  |
| 25.0-29.9 | 1820 (42.8) | 140 (7.7) | 1680 (92.3) |  |
| ≥ 30 | 601 (14.1) | 70 (11.6) | 531 (88.4) |  |
| Diagnosis |  |  |  | < 0.001 |
| OA | 3836 (90.3) | 317 (8.3) | 3519 (91.7) |  |
| RA + AS | 293 (6.9) | 64 (21.8) | 229 (78.2) |  |
| Others | 119 (2.8) | 29 (24.4) | 90 (75.6) |  |
| ASA class |  |  |  | 0.208 |
| 1 | 1734 (40.8) | 158 (9.1) | 1576 (90.9) |  |
| 2 | 2197 (51.7) | 213 (9.7) | 1984 (90.3) |  |
| ≥ 3 | 317 (7.5) | 39 (12.3) | 278 (87.7) |  |
| Anemia |  |  |  | 0.003 |
| Yes | 1188 (28.0) | 140 (11.8) | 1048 (88.2) |  |
| No | 3060 (72.0) | 270 (8.8) | 2790 (91.2) |  |
| Pre-ALB (g/L) |  |  |  | < 0.001 |
| < 35 | 207 (4.9) | 127 (61.4) | 80 (38.6) |  |
| ≥ 35 | 4041 (95.1) | 283 (7.0) | 3758 (93.0) |  |
| Surgical type |  |  |  | 0.474 |
| Primary unilateral | 3662 (86.2) | 349 (9.5) | 3313 (90.5) |  |
| Primary bilateral | 536 (12.6) | 54 (10.1) | 482 (89.9) |  |
| Revision unilateral | 50 (1.2) | 7 (14.0) | 43 (86.0) |  |
| Anesthesia |  |  |  | 0.020 |
| General | 3034 (71.4) | 313 (10.3) | 2721 (89.7) |  |
| Spinal + epidural + CSE | 1214 (28.6) | 97 (8.0) | 1117 (92.0) |  |
| Tourniquet use |  |  |  | 0.118 |
| Yes | 3497 (82.3) | 349 (10.0) | 3148 (90.0) |  |
| No | 751 (17.7) | 61 (8.1) | 690 (91.9) |  |
| Anticoagulant use |  |  |  | 0.937 |
| Yes | 4075 (95.9) | 393 (9.6) | 3682 (90.4) |  |
| No | 173 (4.1) | 17 (9.8) | 156 (90.2) |  |
| TXA use |  |  |  | 0.053 |
| Yes | 2934 (69.1) | 266 (9.1) | 2668 (90.9) |  |
| No | 1314 (30.9) | 144 (11.0) | 1170 (89.0) |  |
| Colloid solution use |  |  |  | 0.941 |
| Yes | 2355 (55.4) | 228 (9.7) | 2127 (90.3) |  |
| No | 1893 (44.6) | 182 (9.6) | 1711 (90.4) |  |
| Drain use |  |  |  | < 0.001 |
| Yes | 3629 (85.4) | 374 (10.3) | 3255 (89.7) |  |
| No | 619 (14.6) | 36 (5.8) | 583 (94.2) |  |
| Transfusion use |  |  |  | 0.055 |
| Yes | 825 (19.4) | 65 (7.9) | 760 (92.1) |  |
| No | 3423 (80.6) | 345 (10.1) | 3078 (89.9) |  |

*Data are reported as number (%); †p-value calculated using Pearson chi-square test or Fisher exact test.

AS, ankylosing spondylitis; ASA**,** American Society of Anesthesiologists; BMI, body mass index; CSE, combined spinal-epidural; HA, human albumin; OA, osteoarthritis; Pre-ALB, preoperative albumin; RA, rheumatoid arthritis; TKA, total knee arthroplasty; TXA, tranexamic acid.
